# Negative Emotion Differentiation Predicts Psychotherapy Outcome: Preliminary Findings

**DOI:** 10.3389/fpsyg.2021.689407

**Published:** 2021-08-02

**Authors:** Gal Lazarus, Aaron J. Fisher

**Affiliations:** Department of Psychology, University of California, Berkeley, Berkeley, CA, United States

**Keywords:** emotion differentiation, dynamic assessment, psychotherapy outcome, patient factors, affect dynamics

## Abstract

Emotion differentiation (ED), the extent to which same-valenced emotions are experienced as distinct, is considered a valuable ability in various contexts owing to the essential affect-related information it provides. This information can help individuals understand and regulate their emotional and motivational states. In this study, we sought to examine the extent to which ED can be beneficial in psychotherapy context and specifically for predicting treatment response. Thirty-two prospective patients with mood and anxiety disorders completed four daily assessments of negative and positive emotions for 30 days before receiving cognitive-behavioral treatment. Depression, stress, and anxiety symptoms severity were assessed pre- and post-treatment using self-reports and clinical interviews. We conducted a series of hierarchical regression models in which symptoms change scores were predicted by ED while adjusting for the mean and variability. We found that negative ED was associated with greater self-reported treatment response (except for anxiety) when negative emotional variability (EV) was included in the models. Probing negative ED and EV’s interactive effects suggested that negative ED was associated with greater treatment response (except for anxiety) for individuals with lower EV levels. Results were obtained while controlling for mean negative affect. Our findings suggest that negative ED can benefit psychotherapy patients whose negative emotions are relatively less variable. We discuss the meaning of suppression and interactive effects between affect dynamics and consider possible clinical implications.

## Introduction

Emotion differentiation (ED), the extent to which emotions are experienced (and labeled) as distinct, has been found to be associated with various positive outcomes (for a meta-analysis, see Seah and Coifman, 2021). It is considered a valuable ability in multiple contexts, providing individuals with essential affect-related information that can guide their behavior in an adaptive manner (Schwarz, 2012; Kashdan et al., 2015). The present study set out to examine whether ED can be beneficial in the context of psychotherapy, and specifically, to what extent those with greater ED respond better to a personalized cognitive-behavioral treatment. We first review findings tying emotion-related constructs to psychotherapy response and then note limitations with their current operationalizations. Subsequently, we explain how ED, obtained using dynamic assessment, can function as a promising predictor of such response.

Patients vary significantly in their response to psychotherapeutic interventions (e.g., Lambert, 2013; Boswell et al., 2016). Traditionally, this variance has been attributed to three classes of factors: treatment factors (Marcus et al., 2014; Firth et al., 2019; e.g., technique), therapist factors (Baldwin and Imel, 2013; e.g., experience), and patient factors (Bohart and Wade, 2013; e.g., personality traits). Among these classes, leading researchers (e.g., Wampold, 2010; Norcross and Lambert, 2011) estimate that a large portion of the treatment response variance can be explained by patient factors, that is, by pre-existing individual differences between patients.

Identifying which patients are likely to respond poorly to treatment and which factors underlie this response can have important clinical implications regarding treatment planning. Such factors can sometimes be addressed by therapists employing specific psychotherapeutic interventions. Moreover, these factors can inform caregivers regarding the intensity of the recommended treatment or indicate a need to employ other treatment modalities (e.g., group psychotherapy and psychopharmacology).

For many years (e.g., Luborsky et al., 1971), clinicians and researchers have attempted to discover specific patient characteristics that are predictive of therapeutic improvement (for review, see Bohart and Wade, 2013). Some characteristics, such as demographic variables (e.g., gender or age), have failed to show consistent associations with therapy outcomes (Cuijpers et al., 2009; Bohart and Wade, 2013). Other characteristics, such as symptom severity (e.g., Firth et al., 2019) or patients’ therapy-related expectancies and motivational factors (e.g., Newman et al., 2006; Constantino et al., 2011), have been identified as more consistent predictors.

Emotional experience, expression, and regulation have all been proposed as key patient factors that can affect psychotherapy outcome (e.g., Greenberg and Safran, 1989; Thoma and McKay, 2015; Fisher et al., 2016), with theoretical and empirical work pointing to the importance of monitoring, processing, and regulating emotions as integral psychotherapeutic process factors (e.g., Pascual-Leone and Greenberg, 2007; Pinna et al., 2020). Though compromised to some extent in many psychopathological conditions (e.g., Dryman and Heimberg, 2018), these abilities are considered vital for patients to be able to benefit from the psychotherapeutic process (e.g., Watson et al., 2011). Specifically, they allow for deeper examination and reflection over one’s experience, creating new meanings, and personally meaningful problem resolution (e.g., Watson and Bedard, 2006; Aafjes-van Doorn and Barber, 2017). In line with these notions, the quality of patients’ emotional processing during sessions was found to be tied to improved treatment outcomes (Pos et al., 2009; Aafjes-van Doorn and Barber, 2017; Pascual-Leone and Yeryomenko, 2017).

Whereas session-based emotional processing and regulation have been shown to be robust predictors of therapy outcome, the predictive validity of pre-treatment emotional processing or regulation indices have been less consistent. In particular, a recent systematic review by Pinna et al. (2020, p. 1) indicated that the links between self-reported alexithymia, an inability to identify and communicate emotions, and treatment outcomes are “complex.” Though in some studies alexithymia was tied to poorer treatment response (e.g., Quilty et al., 2017), this association was absent in others (e.g., Spek et al., 2008).

Additional work has examined other emotional processing or regulation variables as outcome predictors. For example, patients lower in pre-treatment emotion suppression had more favorable treatment outcomes (Scherer et al., 2017; Hosogoshi et al., 2020). Interestingly, in both studies, emotion reappraisal was not predictive of treatment outcome. Lastly, a measure of psychological mindedness, defined as the tendency to turn inward seeking psychological explanations of behavior, people, and problems, has provided mixed results as a predictor of therapy outcome (Bohart and Wade, 2013).

A common limitation shared by most studies addressing the links between patients’ abilities to express, process, and regulate their emotions and psychotherapy outcomes is their attempt to capture dynamic processes using a single-time static intake measurement (Fisher, 2015). This discrepancy severely hinders researchers’ ability to tap the processes underlying patients’ emotional difficulties accurately. The reliance on self-reports for items requiring a high level of reflective capacity further limits the measurement validity.

Though single-time clinician assessment is still considered the gold standard in psychotherapy practice and research, pre-treatment ecological momentary assessment (EMA) is increasingly being employed to assess clinically relevant factors (e.g., Lutz et al., 2018; Rubel et al., 2018; Fisher et al., 2019; Shalom et al., 2020). It allows for intensive repeated measurement of variables of interest and modeling their dynamic inter-relationships (e.g., Fisher and Boswell, 2016). Researchers and clinicians have started to capitalize on EMA’s strengths to explore the extent to which dynamic indices can inform treatment processes and outcomes (e.g., Husen et al., 2016; Lutz et al., 2018; Fisher et al., 2019).

Sophisticated analytic methods can be used to translate such dynamic indices into personalized treatment plans (Fisher and Boswell, 2016; Fisher et al., 2019; Wright and Woods, 2020). At the same time, simpler methods can help identify meaningful individual differences without prescribing specific interventions (e.g., Husen et al., 2016; Lutz et al., 2018; Bosley et al., 2019). Individual differences in the dynamic unfolding of affect (Kuppens et al., 2010) are particularly appealing and relevant in the context of psychotherapy (e.g., Husen et al., 2016).

One affect dynamics index that may be highly informative regarding emotional processing and regulation is emotion differentiation (ED). ED is defined as the extent to which same-valenced emotions are experienced and labeled in a distinct or granular manner (Barrett et al., 2001). Individuals with greater ED tend to represent and describe their emotional states using specific terms (e.g., “enthusiastic,” “irritable,” or “tense”), rather than general or abstract terms (e.g., “good” and “bad”).

Differentiation, particularly between negative emotions, has been tied to various positive outcomes in numerous studies (for a review, see Kashdan et al., 2015; for a recent meta-analysis, see Seah and Coifman, 2021). For example, negative ED has been related to greater self-esteem, lower neuroticism, and less depressive feelings (Erbas et al., 2018; Willroth et al., 2020). Additionally, negative ED was found to serve as a protective factor in the face of various daily stressors (in a community sample; Starr et al., 2017) and of the adverse outcomes of ruminations (in clinical samples; Zaki et al., 2013; Seah et al., 2020). Interestingly, in a recent study (Liu et al., 2020), only a combination of negative and positive ED (but neither independently) moderated an association between trait rumination and increases in depression.

A few candidates have been identified as possible mechanisms underlying the benefits of (mostly) negative ED. ED provides nuanced information about one’s emotions which is likely to translate to more adaptive emotion regulation processes. Indeed, greater negative ED was found to be tied to greater effectiveness of negative emotion downregulation strategies (Kalokerinos et al., 2019). Moreover, affect labeling, the act of putting feelings into words, is widely regarded as a form of implicit emotion regulation technique (for review, see Torre and Lieberman, 2018), and high negative ED individuals are likely to be more accurate and thorough in labeling their emotions. Additionally, greater ED may clarify motivational processes and consequently render the allocation of attentional and behavioral resources more efficient (Kashdan et al., 2015). Lastly, greater ED may involve more accurate causal attributions that rely on better access to the origins of one’s emotional experience. When adverse events are followed by more differentiated and less global emotional states, there is a greater likelihood of identifying the cause one’s emotional response. Accurate attributions are likely to be less *depressive*, that is, less internal, global, and stable (Seligman et al., 1979).

Positive ED, in contrast, has not been tied consistently with adaptive outcomes (despite being moderately correlated with negative ED; Liu et al., 2020). It has been found to be associated with favorable outcomes only under specific circumstances, such as among participants with borderline personality features (Dixon-Gordon et al., 2014) or sub-clinical eating disorders (Selby et al., 2014). In other studies (e.g., Barrett et al., 2001; Demiralp et al., 2012; Kashdan and Farmer, 2014; Willroth et al., 2020), such associations did not emerge, and often they are not being examined or reported.

The impact of ED may be most pronounced and visible in conditions where emotions and their processing play diverse and fundamental roles. Working with patients’ emotions has been identified as a cornerstone of the psychotherapeutic process across theoretical orientations and disorders (Barlow et al., 2011; Greenberg, 2012; Thoma and McKay, 2015). Whereas different orientations may have different foci and employ distinct techniques, they share a primary change mechanism—accessing patients’ emotions and modifying their underlying cognitive-affective mental structures. Patients’ ability to differentiate between their emotions, particularly their negative ones, is probably of great value for such processes.

### The Present Study

The present study expanded recent work regarding the role of dynamic affective patterns (e.g., Husen et al., 2016; Bosley et al., 2019) in patients’ response to psychotherapy by examining the extent to which ED is predictive of treatment outcome. Using EMA conducted prior to a personalized modular cognitive-behavioral treatment (see Fisher et al., 2019) for individuals with generalized anxiety disorder (GAD) or major depressive disorder (MDD), we prospectively estimated patients’ negative and positive ED prior to therapy. These indices were then used to predict patients’ symptomatic improvement from pre- to post-therapy. This study is a secondary data analysis of Fisher et al. (2019) and likewise an extension of Bosley et al. (2019). The latter work examined affect dynamics as predictors of symptoms severity and pre- to post-treatment symptomatic change but did not address ED.

Maladaptive emotional processes are at the core of the development and maintenance of both GAD and MDD. Individuals with GAD often suffer from excessive negative affect that is poorly understood and maladaptively regulated through recurrent worrying (e.g., Mennin et al., 2002). Individuals with MDD often suffer from difficulties identifying emotions, tolerating and accepting negative emotions, and effectively regulating their emotions, and tend to employ maladaptive regulation techniques (e.g., rumination and suppression; for review, see Rottenberg, 2017). These deficiencies in emotional processing are likely to be involved in cognitive biases, such as maladaptive causal attribution processes that play a key role in depression (Peterson and Seligman, 1984).

Given patients’ diagnoses, the characteristics of the treatment, and the nature of ED, we hypothesized that those patients who are better at differentiating between their *negative* emotions would show greater symptomatic improvement. We also examined patients’ ability to differentiate between their *positive* emotions, but we did not expect it to have a similar predictive role for two reasons. First, the evidence for associations between positive ED and adaptive outcomes is much weaker than for negative ED. Second, the context of psychotherapy for depression and anxiety, in which patients work through and around their negative emotions, probably renders their differentiation more meaningful.

Specifically, higher negative ED patients are likely to be more capable of identifying their core maladaptive emotional processes, including inefficient attempts to regulate them (e.g., worrying and ruminating). Moreover, greater negative ED can help patients reinterpret the meaning of negative situations and change maladaptive causal attributions (i.e., internal, global, and stable) that are central to the maintenance of their depressive symptoms. Lastly, the ability of higher negative ED patients to engage in psychotherapy sessions in an emotionally effective manner and regulate their emotions during the sessions is likely to allow for a more focused and efficient therapeutic process.

Notably, following recent work demonstrating limited incremental predictive validity of affect dynamics indices beyond the mean and variability (Bos et al., 2019; Dejonckheere et al., 2019; Wendt et al., 2020), we included these indices in all models. Of note, Bosley et al. (2019) found that the mean and standard deviation (SD) of negative emotions were not associated with treatment response and that the variability of positive emotions was associated with more significant symptom reduction.

## Materials and Methods

### Participants

The current study utilized data from an open trial of personalized modular psychotherapy for depression and anxiety based on the unified protocol (UP; Barlow et al., 2011). In this trial, participants with GAD and MDD completed four daily self-report assessments of affect, behavior, and symptoms for 30-day period prior to treatment. Subsequently, they received psychotherapeutic interventions tailored to their symptom dynamics as assessed during the EMA. A full description of the procedures and outcomes can be found in Fisher et al. (2019).

Individuals with symptoms consistent with possible GAD or MDD diagnoses were recruited from the greater San Francisco Bay Area using referrals, flyers, and internet advertisements. One hundred and seventy-four potential participants passed a brief telephone screening and were invited to an in-person appointment. They underwent a structured clinical interview (the Anxiety and Related Disorders Interview Schedule for DSM-5; ADIS-5, Brown and Barlow, 2014) to verify their diagnosis and assess symptoms’ severity. Inclusion criteria included a primary diagnosis of MDD or GAD, age of 18 to 65 years, and a mobile phone with web access. Exclusion criteria included a history of psychosis or mania, concurrent or recent (within the past 12 months) cognitive-behavioral treatment. Interrater reliabilities for diagnoses (based on video recordings of the structured clinical interviews) were high—GAD and MDD had kappa values of 0.68 and 0.84, and percent agreement of 95 and 92%, respectively.

Fifty-seven individuals (33%) met the inclusion and exclusion criteria, and of these, 40 began treatment. Seven participants withdrew from the study during treatment, and one participant did not complete a post-treatment assessment, leaving 32 participants in the present sample. As shown in Table S1 in the online supplementary material (OSM),^1^ no significant differences were found in demographics, pre-treatment symptoms, and affect variables between participants who completed treatment and post-treatment assessment and those who did not. Twenty of 32 participants in the final sample (62.5%) identified as female, and the average age was 37.9 years (*SD* = 14.3). Sixteen participants (50%) identified as White, nine (28.1%) identified as Asian, four (12.5%) identified as Latino/a, one (3.1%) identified as Black, and two (6.2%) selected “other.” Thirteen (41.6%) individuals were diagnosed with current primary GAD, 5 (15.6%) were diagnosed with current primary MDD, and 14 (43.8%) met the criteria for co-primary diagnoses of both GAD and MDD. Sixteen (50%) participants had at least one current comorbid disorder other than GAD or MDD; these comorbid diagnoses included social anxiety disorder (*n* = 10; 31.2%), specific phobia (*n* = 4; 12.5%), persistent depressive disorder (*n* = 3; 9.4%), agoraphobia (*n* = 2; 6.2%), and posttraumatic stress disorder (*n* = 1; 3.1%).

### Measures

#### Hamilton Rating Scale for Depression

The Hamilton Rating Scale for Depression (HRSD; Hamilton, 1960) assesses the severity of depressive symptomatology. It is a 13-item clinician-administered scale providing severity rating of each overarching symptom cluster on a scale from 0 (not present) to 4 (very severe/incapacitating). The HRSD’s internal consistency ranges from adequate to good (0.73–0.81; Steer et al., 1987; Moras et al., 1992). Its total score interrater reliabilities range from 0.78 to 0.82 (Steer et al., 1987; Moras et al., 1992). HRSD scores correlate strongly with self-report depression measures in clinical samples (Steer et al., 1983).

#### Depression, Anxiety, and Stress Scales

The Depression, Anxiety, and Stress Scale (DASS) is a 42-item self-report questionnaire comprised of three subscales (14 items each) developed to capture levels of anxiety, depression, and stress, as described by the tripartite model (Clark and Watson, 1991; Lovibond and Lovibond, 1995). The anxiety subscale evaluates hyper-arousal unique to some forms of anxiety, and the depression subscale evaluates anhedonia or low positive affect unique to depression. As noted by several researchers (e.g., Holmes and Newman, 2006; Campbell-Sills and Brown, 2010), the DASS stress subscale primarily evaluates tension and irritability prevalent among individuals suffering from GAD. Hence, to create a measure relevant to our entire sample, we combined the depression and stress subscales to be used as the main self-report outcome measure, and used the anxiety subscale as an additional outcome measure. Items were rated on a 4-point Likert scale ranging from 0 to 3 (“did not apply to me at all” to “applied to me very much or most of the time”). In our sample, all three subscales were highly reliable (Cronbach’s alphas for the anxiety subscale were 0.84 and 0.78 for pre- and post-treatment, respectively, for the depression subscale were 0.94 and 0.94 for pre- and post-treatment, respectively, and for the stress subscale were 0.89 and 0.90 for pre- and post-treatment, respectively). The Cronbach’s alphas for the combined depression-stress measure were 0.91 and 0.89 for pre- and post-treatment, respectively.

#### Momentary Affect

For each EMA survey, participants rated their emotional experience over the preceding hours across the survey items using a 0–100 visual analog slider with the anchors “not at all” and “as much as possible.” The surveys included four positive affect items (positive, energetic, enthusiastic, and content) and seven negative affect items (angry, irritable, worthless/guilty, frightened/afraid, down/depressed, worried, and hopeless). Additional items not used for the present study consisted of various symptoms (i.e., loss of interest or pleasure, restless, difficulty concentrating, muscle tension, fatigued, dwelled on the past, avoided people, avoided activities, procrastinated, and sought reassurance). Of note, down/depressed, frightened/afraid, and worthless/guilty were measured as couplets in a single item to reflect the language used in clinical assessment for anxiety or depression, and to prevent patients from being overly exclusive in endorsing them. The within- and between-person reliabilities for the scales were computed using procedures outlined by Cranford et al. (2006). For negative emotions, the within- and between-person reliabilities were 0.81 and 0.77, respectively; for positive emotions, they were 0.82 and 0.58, respectively.

### Procedure

#### Clinical Interview

Following a brief telephone screening, eligible participants were invited to an in-person appointment for a structured clinical interview. The HRSD (along with other measures reported in Fisher et al., 2019) was administered by clinical psychology graduate students supervised by a doctoral-level clinical psychologist.^2^ At this appointment, participants also completed a battery of self-report measures, including the DASS.

#### EMA Surveys

After enrolling in the study, participants’ mobile phone numbers were entered into a secure web-based survey system which prompted participants to answer survey questions four times per day during pre-reported waking hours. During these hours, they received text messages (containing a hyperlink to a web-based survey) approximately every 4 h, with the exact time being randomized within a 30-min window. Each survey expired once a subsequent survey was sent. Participants were instructed to complete surveys for a minimum of 30 days (the total number of days ranged from 29 to 42; *M* = 34.25).

#### Personalized Treatment

Following the 30-day EMA period, participants started modular cognitive-behavioral psychotherapy for mood and anxiety disorders which was personalized *via* the selection of relevant modules from the unified protocol (Barlow et al., 2011) based on the EMA data (Fernandez et al., 2017; Fisher et al., 2019). The average number of sessions delivered in the study was 10.38, ranging from 4 to 14 (mode = 9). Within days of completing treatment, participants underwent an in-person follow-up assessment to evaluate change in diagnosis and symptoms severity. At this assessment, trained graduate students and postdoctoral therapists administered a structured clinical interview, and participants completed various self-report instruments, including the DASS.

#### Data Preparation

Data were processed and analyzed using R (version 4.0.3; R Core Team, 2020). Complete R syntax for the analyses described in this paper is available in the OSM (see footnote 1). Initially, composite positive and negative emotion scores were calculated for each time point of each participant by averaging across positive and negative emotion items. Next, means and standard deviations of these positive and negative emotions composites were calculated for each participant’s time series.

Subsequently, negative and positive ED indices were calculated for each participant using the average consistency^3^ intra-class correlation (ICC; Shrout and Fleiss, 1979), which is a standard procedure (e.g., Erbas et al., 2018). Resulting ICCs were transformed using a Fisher Z-transformation. To ease interpretation, we subtracted the transformed ICCs from 1.00 so that higher values will represent greater differentiation. No negative ICC values were obtained.

#### Data Analysis

To estimate the extent to which ED predicts treatment response, we conducted a series of hierarchical multiple regression models. In the final block, the pre- to post-treatment changes in DASS depression-stress and anxiety scales and the HRSD scores were regressed on (a) ED, (b) affect mean, (c) affect SD (representing emotional variability), and (d) the corresponding pre-treatment outcome measure scores. The means and SDs of momentary affect were included following Dejonckheere et al.’s (2019) recommendations to account for their shared variance with the ED indices.^4^ They were added iteratively to the models after the ED score (and the relevant pre-treatment outcome index) was the only predictor in the first block. All variables were standardized to ease the interpretation of the results. Separate models were estimated for each outcome measure (DASS depression-stress, DASS anxiety, and the HRSD) and for each affective valence (negative and positive emotions). Hence, six models were estimated in total.

To aid the interpretation of significant results vis-à-vis the small sample size, we estimated Bayesian regression models (against an intercept-only null hypothesis) using the BayesFactor package (Morey et al., 2018) parallel to the last steps in the models. For the effects of interest, we present Bayesian credible intervals based on the posterior distribution. Bayesian credible intervals refer directly to the probability of the parameter value to be within the intervals (unlike confidence intervals which refer to the probability of the interval itself to include the true value).

## Results

The total number of observations per participant ranged from 90 to 151 (*M* = 113.19, *SD* = 11.83). The percentage of missing data ranged from 0 to 31.8% (*M* = 12.4%, *SD* = 8.5%). The intercorrelations among the ED indices, affect means, SDs, and outcome variables, as well as these variables’ means and SDs, are presented in Table 1. Among the affect indices, the only significant correlations were between negative ED and negative emotional variability (EV; *r* = −0.50) and between negative and positive EV (*r* = 0.69). Of the correlations between the affect indices and pre-treatment symptoms, the correlations between negative affect mean and DASS depression-stress (*r* = 0.48; notably, correlations with the DASS anxiety and the HRSD were 0.27 and 0.34, respectively) and between negative EV and DASS anxiety (*r* = 0.37)^5^ reached significance.

**Table 1:** Means, standard deviations, and correlations.

S. No.	Variable	*M*	*SD*	1	2	3	4	5	6	7	8	9	10	11
1.	Negative emotion differentiation	−0.13	0.29											
2.	Negative emotion mean	40.61	14.28	−0.01										
3.	Negative emotion variability	13.42	3.56	−0.50^**^	0.00									
4.	Positive emotion differentiation	−0.18	0.27	0.34	0.05	−0.02								
5.	Positive emotion mean	39.24	9.59	−0.16	−0.27	0.01	−0.15							
6.	Positive emotion variability	14.50	4.14	−0.09	−0.25	0.69^**^	−0.29	0.19						
7.	Pre-Tx DASS depression and stress	44.34	13.29	0.27	0.48^**^	0.16	0.28	−0.28	0.09					
8.	Pre-Tx DASS anxiety	12.88	7.81	0.05	0.27	0.37^*^	0.32	−0.18	0.09	0.55^**^				
9.	Pre-Tx Hamilton depression	13.81	3.84	0.03	0.34	0.35	0.11	−0.10	0.20	0.46^**^	0.59^**^			
10.	∆ DASS depression and Stress	−26.28	12.80	−0.35^*^	−0.40^*^	−0.23	−0.18	0.14	−0.32	−0.72^**^	−0.31	−0.45^*^		
11.	∆ DASS anxiety	−7.69	7.10	−0.1	−0.27	−0.37^*^	−0.24	−0.07	−0.26	−0.47^**^	−0.84^**^	−0.56^**^	0.46^**^	
12.	∆ Hamilton depression	−8.03	3.60	−0.17	−0.15	−0.25	−0.01	−0.14	−0.39^*^	−0.19	−0.34	−0.67^**^	0.42^*^	0.56^**^

*Pre-Tx, pre-treatment*.

* *p < 0.05;*

** *p < 0.01*.

Of note, the dependent variable in all models was a change score calculated by subtracting pre-treatment symptoms scores from the post-treatment symptoms scores. Hence, a more positive regression coefficient indicates that the predictor is associated with lower symptom reduction. A more negative coefficient indicates that the predictor is associated with greater symptom reduction.

### Negative ED and Treatment Outcome

#### Predicting DASS Scores

The results of the hierarchical regression models predicting changes in DASS depression-stress and anxiety symptoms by negative affect indices are presented in Table 2’s left and right panels, respectively.^6^^,^^7^^,^^8^ For the depression-stress outcome, when negative ED was the only dynamic index in the model, it did not significantly predict change scores. After introducing the negative EV index, negative ED became a significant predictor with greater differentiation associated with greater symptom reduction. The negative EV index also predicted greater symptom reduction. Lastly, across blocks, higher levels of pre-treatment symptoms predicted greater change. These associations (except the ones with pre-treatment symptoms) did not reach significance in the model predicting anxiety symptoms change.

**Table 2:** Hierarchical linear regressions predicting DASS pre- to post-change by negative emotion indices.

Pred./Outcome	DASS depression-stress					DASS anxiety				
	*β*	*SE*	*t*	*p*	$\eta_{p}^{2}$	*β*	*SE*	*t*	*p*	$\eta_{p}^{2}$
Model 1				*R*^2^:	*0.54*				*R*^2^:	0.72
NED	−0.17	0.13	−1.28	0.211	0.05	−0.06	0.10	−0.59	0.563	0.01
Pre-Tx Sym.	−0.67	0.13	−5.15	<0.001	0.48	−0.84	0.10	−8.52	<0.001	0.71
Model 2				*R*^2^:	*0.55*				*R*^2^:	0.72
NED	−0.18	0.13	−1.36	0.185	0.06	−0.06	0.10	−0.59	0.562	0.01
Mean NE	−0.10	0.15	−0.67	0.511	0.02	−0.05	0.10	−0.46	0.652	0.01
Pre-Tx Sym.	−0.62	0.15	−4.07	<0.001	0.37	−0.83	0.10	−7.96	<0.001	0.69
Model 3				*R*^2^:	*0.63*				*R*^2^:	0.73
**NED**	**−0.40**	**0.15**	**−2.59**	**0.015**	**0.20**	−0.14	0.12	−1.2	0.241	0.05
Mean NE	−0.17	0.14	−1.23	0.231	0.05	−0.07	0.10	−0.64	0.528	0.01
**NEV**	**−0.35**	**0.15**	**−2.38**	**0.025**	**0.17**	−0.16	0.13	−1.26	0.219	0.06
Pre-Tx Sym.	−0.47	0.15	−3.05	0.005	0.26	−0.76	0.12	−6.51	<0.001	0.61
Model 4				*R*^2^:	*0.67*				*R*^2^:	0.75
**NED**	**−0.32**	**0.15**	**−2.07**	**0.048**	**0.22**	−0.09	0.12	−0.73	0.471	0.05
Mean NE	−0.19	0.13	−1.40	0.175	0.07	−0.08	0.10	−0.78	0.443	0.02
**NEV**	**−0.34**	**0.14**	**−2.38**	**0.025**	**0.19**	−0.15	0.13	−1.14	0.264	0.06
NED X NEV	0.24	0.13	1.80	0.084	0.11	0.16	0.12	1.39	0.176	0.07
Pre-Tx Sym.	−0.51	0.15	−3.38	0.002	0.31	−0.79	0.12	−6.77	<0.001	0.64

*Pred., predictor; NED, negative emotion differentiation; Pre-Tx Sym., pre-therapy symptoms; NEV, negative emotion variability; and NE, negative emotion. Using bold font was meant to make significant results more noticeable*.

To further explore the apparent suppression effect, we examined the associations between negative EV and symptoms change scores while adjusting for pre-treatment symptoms scores. These too were not significant, indicating a cooperative (or mutual) suppression (Cohen and Cohen, 1975). Statistically, each of the variables suppressed irrelevant (i.e., residual) variance (in predicting treatment outcomes) in each other. To estimate the size and robustness of the suppression effects, we followed recommendations by Shrout and Bolger (2002), who suggested considering suppression situations as a type of intervening variables models (e.g., mediation; see also Paulhus et al., 2004). Hence, we employed bootstrapping techniques (using the R package lavaan) to calculate the confidence intervals of the “indirect effect,” once with negative EV as the “mediator” and once with negative ED as the “mediator.” The bias-corrected bootstrapped 95% confidence intervals with 10,000 samples were above zero when EV [0.04, 0.53] and ED [0.03, 0.57] functioned as “mediators.”

**Figure 1:**
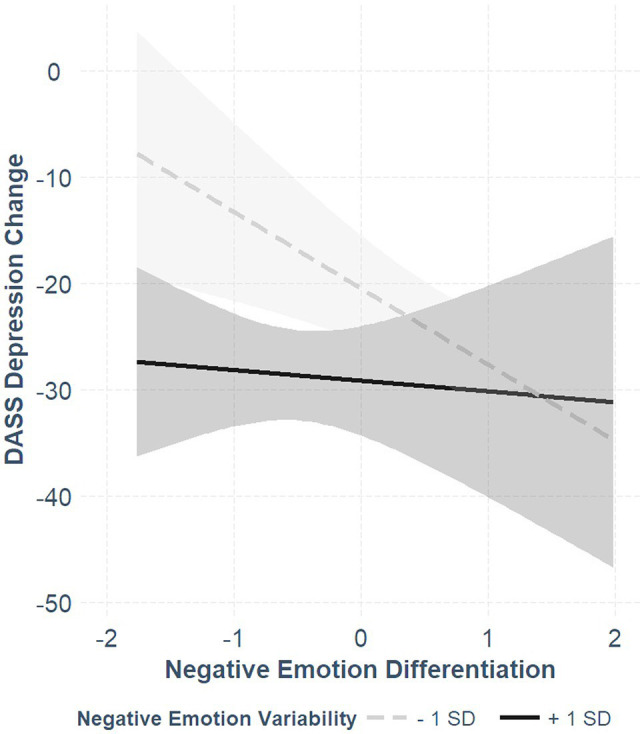
The associations between negative emotion differentiation and DASS depression-stress change scores for high (+1 *SD*) and low (−1 *SD*) levels of negative emotion variability. More negative change scores indicate grater symptom reduction.

Despite the small sample size, we chose to examine the non-independence of negative ED and negative EV by introducing their interaction term into the regression model. As shown in the lower panel of Table 2, the interaction term was below the threshold of statistical significance at alpha = 0.05 (*p* = 0.084) yet had medium effect sizes ($\eta_{p}^{2}$ = 0.11). Moreover, adding the interaction term to the model accounted for an additional 4% of the variance. Hence, we further explored the simple slopes of the associations between negative ED, and symptoms change for different negative EV levels (one SD below and one SD above the mean of negative EV; see Figure 1—the y-axis uses the non-standardized difference scores to ease interpretation). The association between negative ED and the DASS depression-stress was negative and significant for low negative EV (coefficient = −0.56, *SE* = 0.17, *t* = −3.24, *p* < 0.001) and non-significant for high negative EV (coefficient = −0.08, *SE* = 0.23, *t* = −0.34, *p* = 0.74), indicating that for low negative EV, greater negative ED was associated with greater symptom reduction, whereas for high negative EV, it was not. Exploring the simple slopes of EV for different ED levels showed that the association between negative EV and changes in DASS depression-stress was negative and significant for low negative ED (coefficient = −0.58, *SE* = 0.19, *t* = −3.04, *p* < 0.001), and non-significant for high negative ED (coefficient = −0.10, *SE* = 0.20, *t* = −0.34, *p* = 0.63), indicating that for low negative ED, greater negative EV was associated with greater symptom reduction, whereas for high negative ED, it was not.

Given the limited statistical power in the present study, the interaction results should be interpreted with caution. Notwithstanding, in the Bayesian regression model, the empirical means of negative ED and its interaction term with negative EV were −0.28 and 0.21, respectively, only slightly lower than their estimates in the original model. The respective 95% credible intervals were [−0.58, 0.02] and [−0.05, 0.47], respectively. Notably, in none of the models, negative emotion means predicted symptomatic change.

#### Predicting HRSD Scores

**Figure 2:**
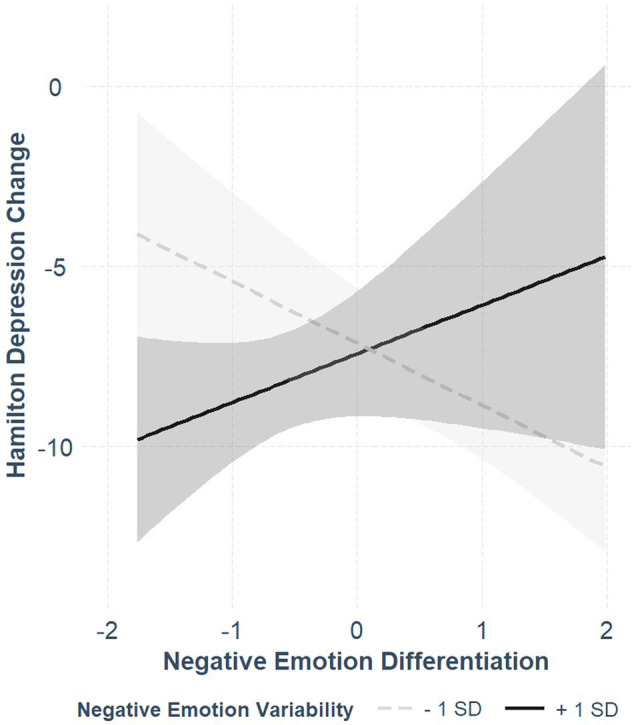
The associations between negative emotion differentiation and Hamilton Depression Rating Scale change scores for high (+1 *SD*) and low (−1 *SD*) levels of negative emotion variability. More negative change scores indicate grater symptom reduction.

The results of the hierarchical regression models predicting changes in the HRSD by negative affect indices are presented in Table 3. Neither negative ED nor negative EV predicted HRSD change scores. Still, following the observed dependency between the predictors, we again added their interaction to the HRSD model. In this model, the interaction term reached statistical significance and accounted for an additional 10% of the variance. We explored the simple slopes (see Figure 2) and found that the effect of negative ED was negative and significant for low negative EV (coefficient = −0.48, *SE* = 0.18, *t* = −2.64, *p* = 0.01), and non-significant for high negative EV (coefficient = 0.38, *SE* = 0.28, *t* = 1.36, *p* = 0.19), indicating that for low negative EV, negative ED was associated with symptom reduction, whereas for high negative EV, it was not. In the Bayesian regression model, the empirical mean of the interaction term between ED and EV was 0.36, and the 95% credible intervals were [0.03, 0.69]. Notably, in none of the models, negative emotion means predicted symptomatic change.

**Table 3:** Hierarchical linear regressions predicting Hamilton Depression Rating Scale pre- to post-change by negative emotion indices.

Pred./Outcome	Hamilton Depression Rating Scale				
	*β*	*SE*	*t*	*p*	$\eta_{p}^{2}$
Model 1				*R*^2^:	*0.47*
NED	−0.15	0.13	−1.11	0.278	0.04
Pre-Tx Sym.	−0.67	0.13	−4.94	<0.001	0.46
Model 2				*R*^2^:	*0.48*
NED	−0.15	0.14	−1.08	0.289	0.04
Mean NE	0.08	0.15	0.54	0.596	0.01
Pre-Tx Sym.	−0.69	0.15	−4.78	<0.001	0.45
Model 3				*R*^2^:	*0.49*
NED	−0.22	0.16	−1.32	0.197	0.06
Mean NE	0.06	0.15	0.40	0.696	0.01
NEV	−0.14	0.18	−0.78	0.445	0.02
Pre-Tx Sym.	−0.64	0.16	−3.91	0.001	0.36
Model 4				*R*^2^:	*0.59*
NED	−0.05	0.16	−0.31	0.755	0.08
Mean NE	0.07	0.14	0.51	0.617	0.01
NEV	−0.04	0.17	−0.25	0.807	0.03
**NED X NEV**	**0.43**	**0.17**	**2.55**	**0.017**	**0.20**
Pre-Tx Sym.	−0.84	0.17	−4.99	<0.001	0.49

*Pred., predictor; NED, negative emotion differentiation; Pre-Tx Sym., pre-therapy symptoms; NEV, negative emotion variability; and NE, negative emotion. Using bold font was meant to make significant results more noticeable*.

### Positive ED and Treatment Outcome

#### Predicting DASS Scores

The results of the hierarchical regression models predicting changes in DASS depression-stress and anxiety symptoms by positive affect indices are presented in Table 4’s left and right panels, respectively. Positive ED did not significantly predict change scores in either DASS depression-stress or anxiety. Positive emotion mean did predict greater changes in anxiety symptoms.

**Table 4:** Hierarchical linear regressions predicting DASS pre- to post-change by positive emotion indices.

Pred./Outcome	DASS depression-stress					DASS anxiety				
	*β*	*SE*	*t*	*p*	$\eta_{p}^{2}$	*β*	*SE*	*t*	*p*	$\eta_{p}^{2}$
Model 1				*R*^2^:	0.52				*R*^2^:	0.72
PED	0.02	0.13	0.16	0.876	0.00	0.04	0.10	0.40	0.692	0.01
Pre-Tx Sym.	−0.72	0.13	−5.37	<0.001	0.50	−0.86	0.10	−8.20	<0.001	0.70
Model 2				*R*^2^:	0.52				*R*^2^:	0.77
PED	0.02	0.14	0.11	0.909	0.00	0.02	0.10	0.18	0.86	0.00
Mean PE	−0.07	0.14	−0.48	0.635	0.01	−0.23	0.09	−2.44	0.021	0.17
Pre-Tx Sym.	−0.74	0.14	−5.26	<0.001	0.50	−0.89	0.10	−9.12	<0.001	0.75
Model 3				*R*^2^:	0.58				*R*^2^:	0.78
PED	−0.07	0.14	−0.53	0.601	0.01	−0.03	0.10	−0.33	0.74	0.00
Mean PE	−0.01	0.13	−0.08	0.941	0.00	−0.2	0.09	−2.16	0.04	0.15
PEV	−0.27	0.14	−2.00	0.055	0.13	−0.15	0.10	−1.55	0.132	0.08
Pre-Tx Sym.	−0.67	0.14	−4.89	<0.001	0.47	−0.86	0.10	−8.72	<0.001	0.74

*Pred., predictor; PED, positive emotion differentiation; Pre-Tx Sym., pre-therapy symptoms; PEV, positive emotion variability; and PE, positive emotion*.

#### Predicting HRSD Scores

The results of the hierarchical regression models predicting changes in the HRSD by positive affect indices are presented in Table 5. No significant effects emerged.

**Table 5:** Hierarchical linear regressions predicting Hamilton Depression Rating Scale pre- to post-change by positive emotion indices.

Pred./Outcome	Hamilton Depression Rating Scale				
	*β*	*SE*	*t*	*p*	$\eta_{p}^{2}$
Model 1				*R*^2^:	0.45
PED	0.06	0.14	0.44	0.661	0.01
Pre-Tx Sym.	−0.68	0.14	−4.91	<0.001	0.45
Model 2				*R*^2^:	0.49
PED	0.03	0.14	0.23	0.816	0.00
Mean PE	−0.20	0.14	−1.46	0.155	0.07
Pre-Tx Sym.	−0.69	0.14	−5.11	<0.001	0.48
Model 3				*R*^2^:	0.54
PED	−0.04	0.14	−0.28	0.783	0.00
Mean PE	−0.16	0.13	−1.18	0.248	0.05
PEV	−0.24	0.14	−1.72	0.097	0.10
Pre-Tx Sym.	−0.63	0.14	−4.66	<0.001	0.45

*Pred., predictor; PED, positive emotion differentiation; Pre-Tx Sym., pre-therapy symptoms; PEV, positive emotion variability; and PE, positive emotion*.

## Discussion

The interest in pre-treatment dynamic assessment based on intensive repeated measurements taken in individuals’ daily life is rapidly growing (Fisher, 2015; Piccirillo and Rodebaugh, 2019; Wright and Zimmermann, 2019; Trull and Ebner-Priemer, 2020), demonstrating the immense potential it holds for clinical science and practice. Such assessment can be used to generate idiographic treatment plans (e.g., Fisher et al., 2019; Wright and Woods, 2020), but can also be employed with a more modest yet important aim of identifying predictors of treatment response. Dynamic assessment is particularly suitable to measure affective processes that unfold in time and reflect individuals’ capacity to process and regulate their emotions. The present work sought to explore one such capacity—individuals’ ability or tendency to differentiate between their emotions.

We estimated ED using an EMA paradigm of 1 month prior to cognitive-behavioral psychotherapy and examined its associations with self-report and clinician-administered outcome measures. Negative ED was found to be negatively associated with negative EV (a risk for multicollinearity problems in the ensuing regression models was largely allayed by low VIF values). Zero-order correlations between negative or positive ED and pre-treatment symptoms did not reach statistical significance. These non-significant correlations may reflect no true correlation in our purely clinical sample, but also the small sample size.

Negative ED was not independently associated with changes in any of the measures. Still, after introducing negative EV into the prediction models, the associations between negative ED and changes in self-reported depression and stress symptoms became significant. Negative EV itself was also not independently associated with change scores, but when concurrently estimated alongside negative ED, it was associated with the depression and stress self-reported change score.

Negative ED and negative EV acted as mutual suppressors increasing each other’s predictive validity once included in the same model (e.g., Paulhus et al., 2004). The shared variance between the two, which underlies the suppression effect, can stem from their common origin in the variance of patients’ momentary affect reports.^9^ Their shared variance may represent their ties with changes in the external contexts patients were exposed to during the EMA period. Greater contextual variability may elicit greater EV and also create the appearance of lower ED (because such changes make it easier for emotions to change together, that is, to be less differentiated). Including ED and EV in the same model allows for examining their effects while taking into account such hypothesized between-patient differences in contextual variability so that purer operationalizations of the processes of interest can be tested. Mutual suppression effects are statistically counter-intuitive yet make much theoretical sense. For example, guilt and shame, which are similar in being “self-conscious,” yet distinct in their objects (the former involves the global self, and the latter involves a specific behavior), were found to act as mutual suppressors in predicting aggression (Paulhus et al., 2004). Excluding self-conscious aggression-irrelevant variance revealed shame and guilt’s “true” predictive power. Future work employing larger samples within different contexts should explore the replicability and generalizability of our suppression finding.

The ED literature contains ample evidence for ED’s independent (i.e., not suppressed) associations with various wellbeing indicators (Seah and Coifman, 2021) and for its protective role in the face of daily stressors or maladaptive behaviors (e.g., Starr et al., 2017; Seah et al., 2020; Nook et al., 2021). ED’s role as a predictor of change processes is yet to be established. However, preliminary findings point to more complex relationships involving interactions between romantic partners’ ED (Lazarus et al., 2021a), between negative and positive ED (Liu et al., 2020), and between ED and personality traits (Oh and Tong, 2020). It seems that associations between ED and changes in outcome measures may be more specific and contingent on other factors.

To further explore the meaning of the non-independence between negative ED and negative EV in predicting self-reported symptoms changes, we added their interaction term to the prediction models. Given the small sample size, the interpretation of these interactions should be made cautiously. The interaction term did not reach statistical significance in predicting the self-report change scores but accounted for a considerable amount of their variance. Hence, we examined negative ED’s effects under different levels of negative EV and found that it was associated with changes in self-reported depression and stress symptoms for low, but not high, level of negative EV. Moreover, in the model predicting clinician-rated change in depression symptoms, the interaction term reached significance, with negative ED being associated with symptoms change only for low levels of negative EV.

Taken together, these findings indicate that pre-treatment negative ED may predict more favorable treatment response for those patients whose momentary experiences of negative emotions are less variable across time. We hypothesize that those patients whose negative emotions are less variable across time have a greater need to differentiate between these emotions due to the persistent or entrenched nature of their negative emotional experiences. Conversely, patients whose negative emotions are more variable across time may be able to benefit from psychotherapy even when they are less capable of differentiating between them. For these patients, their affect and symptomatology may be relatively malleable or plastic. Supporting this hypothesis, Shalom et al. (2020) found that variability in social anxiety symptoms (that include some affective items) before psychotherapy is predictive of sudden gains during the treatment.

Positive ED was not associated with any of the treatment response measures in the current study. Importantly, this finding should not be automatically generalized to other psychopathological conditions or other types of treatments. Positive ED was found to be associated with adaptive outcomes in contexts where positive emotions are prevalent or important (e.g., the transition to parenthood; Lazarus et al., 2021a). From a functional perspective, differentiated experience of positive emotions can aid in eliciting specific and adapted motivational, cognitive, physiological, and behavioral responses to environmental opportunities (Shiota et al., 2014; Beall and Tracy, 2017). In the context of interventions targeting positive affect and reward sensitivity (e.g., Craske et al., 2019), positive ED may prove beneficial.

Of note, the means of patients’ negative and positive emotions throughout the EMA period were not associated with symptomatic change (except for the association between positive emotion mean and changes in anxiety symptoms). These null effects partially echo previous work exploring daily affect and psychotherapy response. Specifically, Husen et al. (2016) found that mean positive and negative daily affect did not predict early response in cognitive-behavioral therapy. In Forbes et al. (2012), mean daily negative (but not positive) affect was tied to a slower rate of symptom reduction during depression and anxiety treatment for children and adolescents. In both studies, greater positive to negative emotions ratio predicted better treatment response (we did not observe a similar pattern in our data). It is notable that the significant predictors of symptomatic change (ED, EV, and positive to negative affect ratio indices) all involve within-person (co)variation, unlike the means, which represent a summary of absolute values. Absolute values may be more liable to various response biases that restrict their efficiency in predicting change scores. While the diverse research contexts and limited sample sizes (*N* = 39 in Husen et al., 2016; *N* = 66 in Forbes et al., 2012) make it difficult to draw firm conclusions, this emergent pattern may strengthen the case for the predictive validity of dynamic indices vs. mean levels.

Identifying patients who fail to sufficiently differentiate between their negative emotions in daily life can guide therapists’ efforts at the first treatment stages. Therapists can employ various techniques and tools developed in the context of leading clinical approaches, including emotion-focused therapy (e.g., Pascual-Leone and Greenberg, 2007) and cognitive-behavioral therapy (e.g., Barlow et al., 2011) to help their patients attain a more differentiated emotional experience. Other capacities, such as mindfulness skills (Van der Gucht et al., 2019), or activities, such as self-monitoring (e.g., Widdershoven et al., 2019), have been shown to improve ED. Patients can then use this newly acquired ability to achieve other therapeutic goals.

### Broader Considerations

This study examined a specific EMA-derived patient factor predictive of treatment response. Current efforts to identify patient factors often adopt data-driven machine learning algorithms that examine large numbers of possible predictors, with the potential to estimate nonlinear associations and higher-order interactions (e.g., Zilcha-Mano, 2019; Webb et al., 2020). Despite the advantages this approach may hold, it suffers from several limitations. First, generalizable findings require very large sample sizes (Archer et al., 2021) often unavailable in psychotherapy context. Second, the resultant models are often a black box with limited interpretability. Third, in the context of psychotherapy outcome prediction, this approach usually relies on self-reports. Arguably, the quality of any statistical model is limited by the quality of the data it includes, and single-time self-reports are inherently limited in their ability to capture dynamic processes representative of prospective patients’ abilities. Due to these limitations, we believe that a theory-driven EMA-based search for specific treatment outcome predictors is necessary and valuable.

A significant advantage of dynamic assessment is that its reliance on associations between repeatedly measured self-report variables helps alleviate the risk of patients being swayed by factors, such as social desirability or experimenter demands typical of single-time self-report assessment (Sened et al., 2018). This risk may be particularly relevant in the context of pre-therapy assessment, where prospective patients may either over (Merckelbach et al., 2019) or under (Warner et al., 2011) report their psychological difficulties and symptoms. Using dynamic within individual patterns as predictors allows researchers to go beyond respondents’ direct awareness and the mean levels of their reports, thus increasing these predictors’ validity.

The discovered interactive effect between ED and emotional variability may suggest that greater attention to interactions between affect dynamics in their relations with other constructs is in place. After all, it is unlikely that these relations follow simple linear regularities, but rather more complex patterns (e.g., Wichers et al., 2015). It is possible that interactions between different dynamic indices will function better than single indices in representing robust interindividual differences. Notably, examining such interactive effects will require increased sample sizes.

In this study, the dynamic indices were derived from surveys collected four times a day, approximately 4 h apart. This data collection scheme was chosen to reduce patients’ burden and provide a representative sample of participants waking hours, but the relatively long measurement intervals run the risk of missing the more rapid affective processes (e.g., Verduyn et al., 2009). An alternative, contextualized approach to dynamic assessment may aim to capture affect dynamics when and where they matter the most, for example, in the vicinity of a stressful event (Dejonckheere et al., 2020; Lapate and Heller, 2020). Moreover, assessment of affect dynamics can be relevant and informative also after psychotherapy has started using either EMA between sessions (e.g., Frumkin et al., 2020) or reports regarding the sessions themselves (Lazarus et al., 2019; Galili-Weinstock et al., 2020).

Lastly, for dynamic assessment of affective processes to reach its full potential, it must involve thorough consideration of the temporal dynamics of the target processes (e.g., Hamaker and Wichers, 2017; Lazarus et al., 2021b). Specifically, a time scale (e.g., Adolf et al., 2021) appropriate for capturing affective changes as they unfold in patients’ daily life should be identified based on prior research (e.g., Verduyn et al., 2009) or theoretical grounds, and dictate the measurement scheme. Additionally, trends (e.g., linear and quadratic; Jebb et al., 2015) and cycles (e.g., diurnal and weekly; van de Maat et al., 2020) should be modeled and interpreted on a case-by-case basis (Fisher and Newman, 2016).

### Limitations

Several limitations of the present study should be acknowledged. First, the available sample size of treatment completers provided low statistical power. Such low power may have prevented us from detecting some effects that would have emerged with a larger sample. This sample size should also suggest caution when interpreting the effects that did emerge, as they may not be generalizable to other samples. Clearly, replications with larger samples are necessary to establish the reported effects’ validity. Of note, the study’s procedure is highly demanding (included both EMA and psychotherapy) and makes larger samples hard to obtain. Moreover, the study did have a large number of within-individual measurements across a prolonged period, increasing the ED indices’ reliability.

Second, though ED was measured prior to treatment, claims regarding its causal role in the treatment should be taken with caution. While we cannot rule out the effects of many “third variables,” the inclusion of pre-treatment symptoms scores in all models narrows this concern somewhat. Future work measuring ED throughout the treatment and at its end can provide further credibility for causal inferences.

Third, the items used to estimate patients’ ED suffered from two limitations stemming from the original focus and the purpose of the data collection. The PED measure was based on only four positive emotions and included one unspecific item (i.e., “positive”). This narrow measurement was meant to reduce participants burden but might have crippled our positive ED index. Given the growing interest in the role of distinct positive emotions (e.g., Weidman and Tracy, 2020), future dynamic assessment work should operationalize positive ED using a larger number of items. The NED measure was partially based on items with conjoint terms. Two of these items (i.e., down/depressed and frightened/afraid) were used to avoid patients being overly exclusive in endorsing them; the third (worthless/guilty) was used as an adaptation of one of the key DSM depression symptoms and involves clearly two distinct emotions. These issues might have added noise to our NED measurement. Specifically, endorsement of the same value for these items on different occasions may reflect different experiences for patients who could differentiate between the conjoint terms. Consequently, the ED scores of these patients might have been underestimated. The usage of such items is particularly problematic when studying ED because how individuals interpret them is a derivative of ED itself and thus runs the risk of leaving important between-individual variability in ED unaccounted for. Furthermore, the negative affect measurement included a limited number of items, and future work is necessary to assess whether findings generalize to differentiation among other emotions.

## Conclusion

The present work took a preliminary step in demonstrating the utility of dynamic assessment to identify affect-processing patient factors predictive of treatment outcome. We found that negative ED predicted better treatment response when emotional variability was taken into account. Our findings suggest that negative ED may play an important role in the success of psychotherapeutic interventions.

## Data Availability Statement

The original contributions presented in the study are included in the article/supplementary material; further inquiries can be directed to the corresponding author.

## Ethics Statement

The studies involving human participants were reviewed and approved by the Committee for Protection of Human Subjects (CPHS), University of California, Berkeley. The patients/participants provided their written informed consent to participate in this study.

## Author Contributions

GL developed the idea for the study and conducted the data analyses and interpretation under the guidance of AF. AF performed the data collection. GL drafted the paper under the guidance of AF who provided critical revisions and comments. All authors contributed to the article and approved the submitted version.

## Conflict of Interest

The authors declare that the research was conducted in the absence of any commercial or financial relationships that could be construed as a potential conflict of interest.

## Publisher’s Note

All claims expressed in this article are solely those of the authors and do not necessarily represent those of their affiliated organizations, or those of the publisher, the editors and the reviewers. Any product that may be evaluated in this article, or claim that may be made by its manufacturer, is not guaranteed or endorsed by the publisher.

## Footnotes

^1^ https://osf.io/vqsdb

^2^Inter-rater reliability was calculated only for the ADIS-5 diagnoses.

^3^We opted to use the consistency index that ignores reported items’ means, as we were concerned the latter may reflect response tendencies and not true differentiation. The correlations between the consistency and absolute agreement indices were 0.95 and 0.96 for the negative and positive indices, respectively.

^4^Due to the small sample size we opted not to include additional affect dynamics indices.

^5^We avoided noting the simple correlations with the difference scores because the associations between the pre-treatment symptoms and (a) post-treatment symptoms (*r* = 0.42, 0.43, and 0.47 for DASS depression-stress, DASS anxiety, and the HRSD, respectively) were moderate and significant, and (b) the affect indices were non-zero. These associations make difference-scores correlations hard to interpret (Allison, 1990).

^6^We reran all models while adjusting for gender, age, and number of EMA surveys completed. Results remained essentially unchanged.

^7^To allay the concern of multicollinearity, we examined the variance inflation factor (VIF) of the predictors in all models. All the values were below 1.86, indicating that the models did not suffer from a multicollinearity problem.

^8^At the request of a reviewer, we examined additional models predicting each subscale separately and a model predicting the DASS total score. The results of these models are presented in Tables S2 and S3 in the OSM. As can be seen in Table S2 (NA indices), whereas no significant effects emerged for the separate DASS subscales, effects parallel to the combined DASS depression-stress measure emerged for the DASS total score. As can be seen in Table S3 (PA indices), no significant effects emerged for any of the outcome variables.

^9^Whereas EV represents the variance in negative affect reports attributable to differences between measurements, ED represents the variance in affect reports not attributable to differences between measurements and between specific items’ means (that is, their inconsistency).
